# Persistence and Innovation in the Greco-Roman Medical Tradition: The Reading and Writing Practices of a Tenth-Century Monk

**DOI:** 10.1093/shm/hkaf032

**Published:** 2025-07-02

**Authors:** Silvia M Marchiori

**Affiliations:** Department of History and Philosophy of Science, University of Cambridge, UK

**Keywords:** early medieval medicine, medical miscellanies, ancient Rationalism, zodiac wheel

## Abstract

By offering an organic reading of the tenth-century medical miscellany BnF, Lat. 7028, this article questions assumptions about the erratic and rudimentary nature of early medieval medicine, highlighting the compiler’s purposeful selection and manipulation of contents. Following the tenets of the ancient sect of Rationalists, as described in Celsus’ *De medicina*, the compiler gathered a consistent yet non-linear compendium, blending texts about the mythological Greek origins of medicine, anatomical parts, natural philosophy and different sets of therapeutical options, encompassing regimen, medications, and surgery. The Greek-Latin monk Johannes Philagathos is arguably the intellectual author of this eclectic miscellany, which he assembled thanks to networks of people and books that circulated between Byzantine and Ottonian areas. While preserving ancient and late antique medical traditions and visual models, this manuscript witnessed the reception of medicinal drugs from eastern lands and their inclusion in recipes, a few centuries before the flourishing of the School of Salerno.

In 190 nicely written parchment sheets, which correspond to the skin of circa 50 sheep and endless days of backache, a tenth-century monastic compiler selected and copied out medical information from more than a dozen different sources. From the widely known *Etymologies* of Isidore of Seville and the late antique *Ars medicinae*, he copied Greek myths on the birth of medicine, the life of Hippocrates, and the medical deontological code, with instructions on how physicians should appear and behave. Then, after providing a list of anatomical definitions, he copied out a large portion of Cornelius Celsus’ *De medicina*, a first-century CE text that circulated in northern Italy in a handful of manuscripts at this time. Next came four different sets of recipes for medicinal oils, pessaries, compounds, and a detailed list of simples. After this, our compiler turned to studying the properties of stones with the help of Isidore’s *Etymologies*, again, and a Hellenistic lapidary attributed to Damigeron. This section was followed by a list of the 21 virtues of substances from the pseudo-Galenic *De dynamidiis*, fragments on sphygmology and urinoscopy from the hybrid *Liber Anathegore de pulsis et urinis*, and further Celsian extracts, combined with the spurious *Liber cyrurgie Ypocratis*, to describe simple surgical operations for bloodletting. In this last section, the compiler left blank spaces after each operation for images, which he never drew. Yet, he completed two beautiful circular diagrams in the form of *rotae*, which portrayed two major agents of influence on the body, namely, the zodiac signs and the winds.^1^

## Mistreated, mishandled, misused

The medical miscellany described above is now in Paris at the Bibliothèque nationale de France, shelf mark Lat. 7028, hereafter referred to as codex Parisinus. Around 1000, such miscellanies, primarily compilations of ancient and late antique texts, were the most common form of medical literature.^2^ Compared to the philosophical sophistication of Galenic medicine, early medieval medical theory mainly focused on prognostics, regimen, and *materia medica*, rather than aetiology and diagnosis. Deprecatory narratives of the early Middle Ages have claimed that compilers revealed no clear understanding and predetermined purpose during their scribal activity, which resulted in the production of erratic, rudimentary, and superstitious medical accounts.^3^ In contrast, Faith Wallis argued that compilers purposefully organised their activities and pursued specific agendas.^4^ Peregrine Horden has similarly defended early medieval medicine, noting the problematic interpretation of medical miscellanies, but offering a syncretic view of early medieval science, religion, and magic.^5^

Codex Parisinus belongs to the tradition of medical miscellanies that hinder a linear reading of the contents, as it consists of scattered quires that address different subjects, escaping any apparent logical order. Yet, despite the manuscript’s incomplete nature and form, the compiler produced an organic assortment of medical texts in a multi-text monoblock compilation, or homogenous collection.^6^ While occasionally mentioned in different studies about early medieval medicine and cosmology, this manuscript has never been studied as a cohesive textual unit. In the line of recent appreciations of miscellanies by scholars like Anna Dorofeeva, Marilena Maniaci, and Michael McVaugh, this article aims to provide an organic reading of this textual unit and to show that the selected contents of Parisinus, all penned by a single-hand, reveal very specific interests and disclose the reading and writing practices of a tenth-century erudite monk.^7^

This article investigates the compiler’s intellectual process in collecting information with a preference for texts that comply with the tenets of ancient Rationalism, a medical sect that developed in fourth-century BCE Greece and was embraced by prominent physicians such as Diocles, Praxagoras, Asclepiades, and partly Galen.^8^ In the centuries-long historical arch of the unfolding of Rationalism, codex Parisinus represents a transitional codification of this medical and epistemological doctrine, and it provides a prehistory to the early modern philosophical evolution and reappraisal of Rationalism, which relied on classical sources like Galen and Celsus.^9^ In this thematically cohesive manuscript, such a Rationalist orientation questions assumptions about the putatively primitive state of early medieval medicine, revising the common understanding of pre-Salernitan medicine.^10^

By focusing on cardinal parts of the manuscript that concern the reception of ancient histories of medicine, traces of medical Rationalism, and therapeutical strategies, this article challenges the negative connotation of pre-Salernitan medicine. While widely used in literature, the adjective ‘pre-Salernitan’ is not a useful analytic term for early medieval medicine. It flattens the multifaceted local dynamics of medical learning and practice that developed in monasteries, cathedral schools, and lay society, where medicine and spirituality converged in manifold practical articulations.^11^ It also overemphasises the importance of Constantinus Africanus’ translations and the School of Salerno in the renewal of learned medicine. Charles Burnett, Danielle Jacquart, Erik Kwakkel, Francis Newton, and others have explored the exceptional story of Constantinus, who translated medical Arabic texts in the late eleventh century and disseminated new knowledge thanks to the scribal activity in Monte Cassino.^12^ The reception of newly translated Aristotelian, Galenic and Arabic texts undoubtedly affected western medicine, providing impetus for the development of Scholasticism, but Arabic-oriented narratives reiterate the *topos* of the Dark Ages, the barbaric West, and the isolated occurrence of a twelfth-century Renaissance.^13^ The case of codex Parisinus, instead, shows that ancient learned medical texts kept circulating between the decline of the late antique medical school in Ravenna, often depicted as the swansong of the Alexandrian tradition, and the rise of the Salernitan School, with its attention to theoretical questions, natural philosophy, and new pedagogical tools for teaching medicine.^14^

Partly because of the focus on Monte Cassino and Salerno, the role of Byzantium in the transmission of ancient and Arabic medicine has come to be appreciated only in recent years, when scholars like Matteo Martelli, Petros Bouras-Vallianatos, and others revealed the dense networks of intellectual and commercial relationships that connected Byzantium with Arabic, Jewish, and Latin communities, highlighting the transcultural nature of medieval Mediterranean medicine.^15^ Along these lines, the contents of codex Parisinus mirror the dual identity of its compiler. By examining the manuscript’s features and the geographical scope of its production, this article supports previous identifications of the intellectual author as the Greek-born Latin monk Johannes Philagathos, member of the Ottonian court, prominent churchman, and later antipope.^16^ Philagathos’ hybrid Greek-Latin identity allowed him to act as a political and intellectual agent at the crossroads of continental Europe and the Byzantine area of influence, which included southern Italy until the eleventh century. With a somewhat eclectic, yet firmly Rationalist-oriented mind, he collected multiple texts to shape an original and elaborate account of medicine that encompassed ancient Greek myths, celestial diagrams with eastern Mediterranean iconographical models, and the reception of exotic *materia medica*.

## Following the tracks

The author of codex Parisinus left only one personal trace in his manuscript, namely, a short poem at the end of the first extracts from Celsus’ *De medicina*.^17^ Comparing himself to a fisherman who returned to land with a big catch, *sacer Iohannes* claimed that, with great effort, he corrected part of the book at the request of his pupil. The last verse ‘I did what I could; what I could not, reluctantly, I omitted’ is a sophisticated calque of Ovid’s verse ‘I will hate if I can; if not, reluctantly, I will love’, which displays the compiler’s creative reliance on his erudite readings.^18^ As previously mentioned, the identity of *sacer Iohannes* has been linked to the figure of Johannes Philagathos (940s?-1001/13).^19^ Born in Rossano, a vibrant centre of Greek culture, monasticism, and scribal activities in the Calabria region in southern Italy, Philagathos (from the Greek, ‘lover of the good’) was described in other sources as Calabrian (*calabritanus*), Greek Johannes (*Iohannes Graecus*), and of the Greek nation (*natione graecus*).^20^ He was likely bilingual in Latin and Greek, and in 983, he entered the imperial court, earning the trust of the emperor Otto II and his consort, the Byzantine princess Theophano, and becoming tutor to their son, the future Otto III. In those years, while acting as Otto II’s chancellor, Philagathos established a close relationship with Theophano, who often relied on him in the years of her regency before Otto III’s coronation as emperor.^21^ The tenth-century Ottonian court displayed interest in Greek cultural and artistic traditions, even though Theophano’s role in the introduction of Greek customs at court is still disputed.^22^ Nevertheless, Byzantine culture shaped the intellectual profile of Otto III, who famously asked the learned priest Gerbert of Aurillac to help him replace Saxon vulgarity with Greek subtlety.^23^ Arguably, Philagathos could have compiled codex Parisinus to gift it to his pupil (*alumpnus*) mentioned in the short poem, probably the young Otto III.

Evidence of Philagathos’ interest in medicine is found in an inventory of his library in Piacenza, a city midway between Milan and Parma, where he was appointed archbishop in 988. In addition to two books of Livy, Persius’ satires, the works of the theologian Orosius, two capitularies, a copy of Fulgentius’ writings with a text on orthography under the name of Isidore, two glossaries, and Boethius’ commentary on Porphyry’s *Isagoge*, Philagathos’ collection included a medical compilation (*medicinalem unum*) that might correspond to codex Parisinus itself.^24^ As an erudite courtier, Philagathos actively contributed to the development of the Ottonian library, and his learning was highly esteemed.^25^ When Otto II appointed him abbot of the Benedictine abbey of Nonantola, near Modena, in 982, the official diploma referred to Philagathos as archimandrite, which designated the leading figure of one or more monasteries in the Byzantine Empire, also praising his erudition, Greek knowledge, and alleged holiness.^26^

Additional evidence from a different manuscript supports Philagathos’ interest in medicine and connects the production of codex Parisinus to the abbey of Nonantola.^27^ Manuscript Vatican Library, Vat.lat. 5951 (hereafter referred to as V) is a ninth-century copy of almost the entire eight books of Celsus’ *De medicina*, together with a short twelfth-century gynaecological excerpt. Despite the unclear specification of its site of production, it has been linked to Nonantola by a marginal invocation to Saint Sylvester, patron of the abbey^.^^28^ This manuscript was the source of the Celsian text copied out in codex Parisinus, and it bears annotations by a medieval reader, arguably a contemporary of the scribe of Parisinus that was familiar with Philagathos’ medical activity.^29^ These annotations include two marginal recipes with instructions for the preparation of a remedy for coughing and dysentery and a mixture for incense.^30^ The recipe for coughing is attributed to *Iohannes calaber*, and it states: ‘pill experimented for coughing and for people who suffer from dysentery of the Calabrian Johannes’, a title that matches with Thietmar’s chronicle, where he called Philagathos *Iohannes calabritanus*.

During his years as abbot of Nonantola, Philagathos operated in an area that maintained connections with Byzantium.^31^ In late antiquity, Nonantola was located in a traditionally Byzantine area, the Exarchate of Ravenna and material evidence indicates exchanges between Byzantine lands and Nonantola in the early Middle Ages. Uncommon Byzantine ceramics were found at the tenth-century site of the abbey, and exquisite vestments of eastern origin are still displayed in the abbey’s treasury.^32^ In addition to goods, visits of Greek monks from southern Italy were also fairly common. The priest and scholar Cosma of Matera, for instance, worked in the abbey of Nonantola in the early eleventh century and made translations of religious texts from Greek into Latin.^33^ In the tenth century, the abbey housed a library and a *scriptorium*, and it was at the centre of long-distance networks of people and books that connected northern Ottonian Europe and the southern Byzantine and Arabic Mediterranean.^34^ Manuscripts produced in Nonantola show stylistic influences both from the area of Monte Cassino, which provided the earliest core of the library, and from northern centres like Verona and Bobbio, which belonged to the imperial area of influence.^35^

While interacting and exchanging manuscripts with abbeys in Corbie, Saint Denis, and Tours, but also Reichenau, Saint Gall, and Fulda, Nonantola had a special role in maintaining formal relationships between western monarchies and the Byzantine Empire.^36^ In 813, Nonantola’s abbot Petrus went to Byzantium as Charlemagne’s legate together with Reichenau’s abbot Heito and the bishop of Trier Amalarius, and Charlemagne’s grandson Lothair I sent Nonantola’s abbot Ansfried in 828.^37^ Similarly, Philagathos’ career reached its peak when Otto III sent him to Byzantium to negotiate his marriage with a Greek princess in 995. The mission failed, and soon after his return, Johannes and Crescentius II Nomentanus, a Roman nobleman who sought the support of Byzantium, conspired against Pope Gregory V, Otto’s cousin and ally. In 997, Johannes was illicitly appointed Pope John XVI, thus becoming an antipope. The following year, while escaping the avenging expedition led by his former pupil, now emperor, he was captured, blinded, maimed, publicly humiliated, and imprisoned, possibly in a Roman monastery, or in Fulda, where he died in the early eleventh century.^38^

## Greek erudition and tailored Rationalism

While the dubious dating of codex Parisinus and the absence of other revelatory marginal annotations hinder a conclusive identification of Johannes Philagathos as the author, this analysis of the manuscript stands on its own as the original creation of a Benedictine monk who was an expert in medicine and Greek language, with connections to Byzantium and a strong Rationalist learning. The introduction of the manuscript clearly displays Greek features, providing evidence of the uninterrupted circulation of Greek sources in Byzantine southern Italy, their reception in monastic libraries, and their survival in forms that do not display Christianising manipulations, as instead happened in some Carolingian medical manuscripts.^39^ Furthermore, the compiler intervened in the introductory texts to tailor his account of the history of medicine to the tenets of the ancient medical sect of Rationalists. Ancient Rationalists, or Dogmatists, argued that physicians should establish chains of causation to explain disease and prescribe treatments, relying on their in-depth knowledge of hidden and evident causes, the anatomy of bodily parts, and their internal functioning, which required a broad understanding of natural philosophy. They paired theoretical frameworks to explain the body’s nature and its functioning with experiential knowledge derived from everyday practice, challenging the neat separation of intellectual and experiential epistemic processes, but supporting the primacy of deductive thought.^40^

In order to provide information about the Greek origins of medicine and the prestigious figure of Hippocrates, our compiler copied a widely circulating excerpt from Isidore’s *Etymologies* IV.ii on the inventors of medicine and the so-called *Ars medicinae*, a compilation of texts that had Greek origins and survived in medieval Greek, Latin, and Arabic versions. The *Ars medicinae* often circulated together with the Hippocratic *Law* and *Oath*, and with the short text *How should the physician be* (*Qualis debet esse medicus*), as in the case of our codex. While other manuscripts preserve only excerpted versions, Parisinus bears the earliest known complete Latin version of this Hippocratic collection, which concerns the origins of medicine in ancient Greece, the myth of Asclepius, the historical legend of Hippocrates and the medical deontological code, with a detailed description of how physicians should present themselves and behave.^41^

Textual hints clearly reveal the Greek origins of this version of the *Ars medicinae*. To narrate Asclepius’ death at the hands of Zeus, the text mentioned the Greek tragedy *Alcestis* by the fifth-century BCE dramatist Euripides.^42^ Copies of his tragedies were extremely rare in the Middle Ages, and they survived only in a handful of Greek manuscripts in the Byzantine Empire. Ancient and medieval translations into Latin did not exist, and this unparalleled reference to Euripides stands out as a learned reference hardly recognised by the compiler. Shortly later, the iconographical description of Asclepius betrays an improper translation from the Greek. The text describes a bearded Asclepius, sitting with his daughter Hygieia, and holding an egg ‘because there is no expertise without transformation’, a bowl, presumably filled with a healing potion ‘for the cries of people in pain’, and a pomegranate, which represents the multiple habits of people.^43^ According to the text, these attributes were most frequently related to Asclepius ‘in odiis capitaneis’, which literally means ‘in the chief hatreds’ in classical Latin, but likely meant the famous tales of Greek poets, signalling the assimilation of the Greek word for ode (ὠδή) into the Latin vocabulary (*oda*).^44^

Other details of the *Ars medicinae* make this account unique when compared to other sources. Tracing the descendance from Asclepius to Hippocrates, this text reports that once he left his motherland Cos, Hippocrates travelled to Athens during a plague outbreak. Hippocrates happened to observe that blacksmiths and other artisans who worked with fire were immune to the plague, and therefore, he ordered that huge bonfires be lit to purify the noxious air. This account of the story of Hippocrates in Athens is peculiar, and as far as it is known now, it appears only in this manuscript. This version displays significant Empiric nuances, as Hippocrates discovered how to cure the plague by the chance observation of blacksmiths rather than by means of theoretical speculation. Another more Rationalist version survived in the writings of Pliny, Galen, and Aëtius of Amida, who wrote that Hippocrates understood the hidden cause of the plague, namely, the noxious constitution of air, only by reasoning.^45^ The compiler, however, did not remark on this difference between Rationalist and Empiric epistemologies, and he did not emend this passage. An implicit, yet evident ideological intervention in the text, instead, concerns the last part of the *Ars medicinae*, which lists ancient physicians that belonged to the three medical sects of the Rationalists, the Empirics, and the Methodists. In codex Parisinus, the names of Empiric and Methodist physicians are omitted, and the resulting list implies that Hippocrates’ successors exclusively belonged to the Rationalist sect.^46^ While it is possible that the compiler relied on an incomplete version of the text, an organic reading of the entire manuscript suggests that Johannes actively manipulated his sources to support the Rationalist sect.

After a short text on anatomical definitions, Rationalism infiltrates again codex Parisinus in an excerpt from *De medicina*, an intrinsically practical text by the first-century CE encyclopaedical writer Aulus Cornelius Celsus, who designed it to explain Greek medicine to Latin readers.^47^ In the long proem, Celsus extensively described the three major sects of ancient medicine, evenly outlining their different epistemological tenets, therapeutical strategies, and the names of relevant members. Johannes likely accessed Celsus’ *De medicina* in the previously mentioned manuscript Vat.lat. 5951, which bears an easily readable copy of the entire proem. In his selection of contents, however, the compiler copied only the very end of the proem, where Celsus expressed his preference for a sort of mild Rationalism, writing:

Therefore, to return to what I myself propound, I am of opinion that the Art of Medicine ought to be rational, but to draw instruction from evident causes, all obscure ones being rejected from the practice of the Art, although not from the practitioner’s study.^48^

In disagreement with stricter Rationalists, Celsus stated that the ideal physician should know the basics of natural philosophy and hidden causation but held that this information was not useful in medical practice, which should focus on understanding evident causes like regimen and winds, as previously explained in the proem.^49^ The compiler of codex Parisinus followed these recommendations in his selection of medical topics. The structure of the whole manuscript mirrors the Celsian understanding of therapeutics, as divided into dietetics, pharmacy, and surgery, a tripartition already mentioned in the first part of the *Ars medicinae*.^50^ Johannes fashioned his medical account diligently following these ancient ideals of therapeutics, as he included texts on diet and regimen, extensive collections of recipes, excerpts from Celsus’ surgical books, and instructions on how to perform cauterizations.

The compiler’s manipulation of Celsus’ work went beyond the proem. Despite its extremely scarce circulation and use in the Middle Ages, *De medicina* consisted in an invaluable source of ancient medical lexicon in original Greek forms. In comparison to the two extant late antique copies of *De medicina*, our compiler drastically altered Celsus’ work by extrapolating and reassembling information, but most importantly, he often revised the orthography of Greek words extant in manuscript V.^51^ Johannes transliterated Greek words spelt in Latin letters, so that, for instance, *ptiriasi* and *hypocrisin* became ΠΤΙΡΕΙΑΣΙS and YΠΟΚΡΙΣΙΝ.^52^ Instead of copying ΑΔΟΔYΝΑ (*adodyna*) for the word *anodyna*, a name for analgesic pills, he erroneously corrected it with ΑΝΟΔΕYΝΑ.^53^ In another section, the compiler gave his own name to a sleeping pill by using a Greek name for narcotics, *diacodion* (from δια + κωδύα , poppy head), revealing his familiarity with the ancient pharmacological vocabulary.^54^ While engaging in practices of textual emendation, this highly learned compiler also selected from *De medicina* information on healing treatments. Without any knowledge of natural, non-natural, and preternatural elements that influence health and disease, which circulated later in a compendium on Galenic medicine by H.unayn ibn Isḥāq, known in the Latin West as Johannitius, our compiler focused on bodily constitutions and external factors of health and diseases, namely food, drink, physical activity, the seasons, and the weather. In particular, the compiler devoted attention to different kinds of food, excerpting extensive instructions from *De medicina* I-III on how to fatten and thin, heat and cool, dry and moisten the body, physical exercise, and different needs and diets according to age and season. Johannes also copied long lists of Celsian recipes for emollient unguents, eyedrops, pills, and plasters to cure ophthalmia and toothache among other conditions, as well as beauty secrets to hide pimples and freckles.^55^

Codex Parisinus also includes two surgical sections partially extracted from Celsus’ work. Of the 33 chapters in *De medicina* VII, which concerns surgical procedures, Johannes copied only four, regarding abscesses, anal fistulas, the extraction of missiles, and simple oropharyngeal procedures like tonsillectomy and cutting the frenulum; however, he devoted much attention to remedies for broken bones described in book VIII. The Celsian surgical procedures are complemented by the *Liber cyrurgie Hypocratis*, a pseudo-Hippocratic set of instructions on how to perform cauterisation that likely belonged to the writings of the first-century CE Egyptian-Greek surgeon Heliodor.^56^ The compiler prefaced the *Liber cyrurgie* by excerpting a passage from Celsus’ *De medicina* VII on the qualities of the ideal surgeon, who is described as young, strong and steady, ambidextrous, and keen-sighted.^57^ Heliodor’s text provided instructions on how to perform bloodletting with the aid of a cautery, and the compiler organised the text in short sections separated by ample blank spaces, as he arguably intended to draw images or to have them added by an illuminator, as extant in other medieval manuscripts.^58^ Other examples of medieval herbals designed to include images that were never drew might suggest that these manuscripts were copied in haste, or that they were moved from the *scriptorium* before their completion.^59^ The reason for the absence of images in codex Parisinus is difficult to conjecture, especially considering that the manuscript bears two detailed drawings, which are addressed in the following section. Given the incomplete nature of the manuscript, however, it is possible that the compiler’s itinerant life and later political turmoil hindered the completion of these drawings at his own hands or those of another monastic scribe.

## A dual understanding of celestial influence

If the previous section showed that the manipulation of the *Ars medicinae* and the excerpts from Celsus’ work, together with the selection of topics, reflected Rationalist tendencies, this line can also be perceived when focusing on the manifold therapeutical strategies offered in the manuscript. In different sections, the compiler examined an important kind of evident cause of disease according to ancient Rationalism, namely, the winds. Embedded within the tradition of the Hippocratic *Epidemics*, *Prognostics*, and *Airs, Waters, Places*, winds had for long been considered extrinsic causes of disease, and they were later included among the six non-natural elements in H.unayn ibn Isḥāq’s ninth-century introduction to Galenic medicine.^60^ By juxtaposing two different ways of understanding heavenly influence, the one focusing on zodiac signs and the other on winds, the compiler expressed his distrust in the former and tacitly argued that among celestial causes, winds were much more important than astrological influence.^61^

**Fig. 1:**
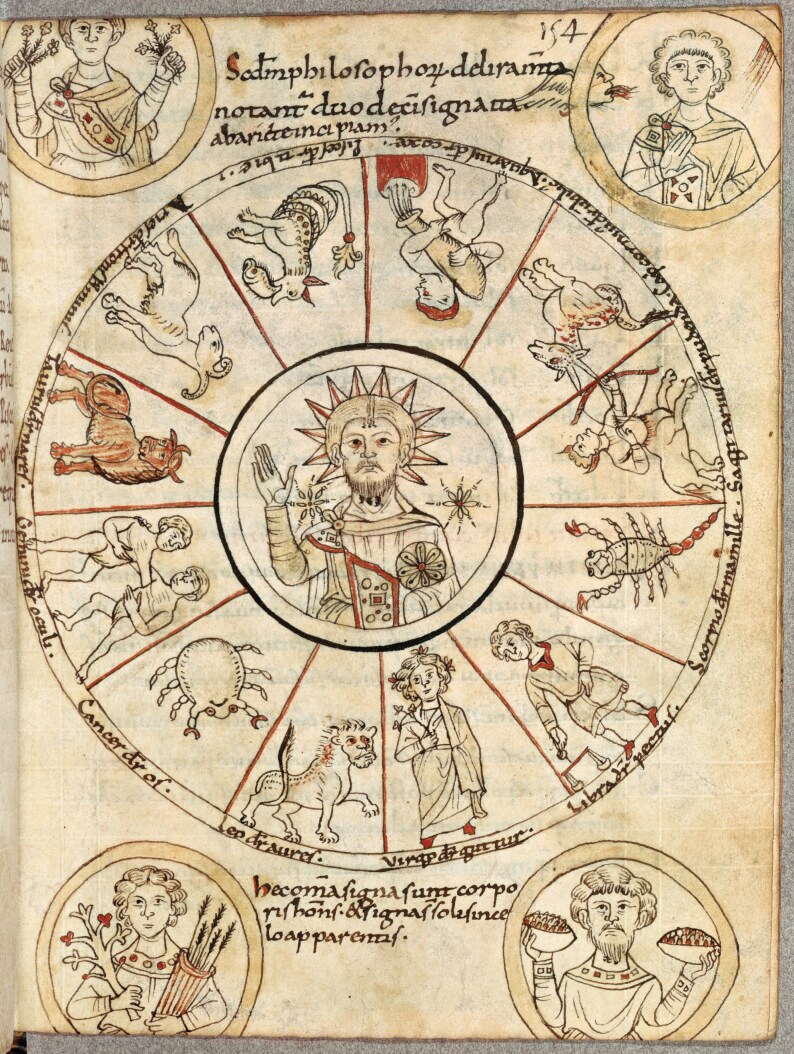
*Rota* with the zodiac signs. Ms Paris, BnF, Lat. 7028, 154r. Courtesy of the Bibliothèque nationale de France.

In order to visualise this twofold connection of celestial agents to terrestrial bodies, and to link the macrocosm to the microcosm, two wheel-shaped diagrams, or *rotae*, support the textual information extracted from Celsus’ and Isidore’s works.^62^ The first *rota* (Figure 1) represents the 12 zodiac signs running counter-clockwise. In the middle, the image of the sun recalls the iconography of Helios, Mithra, and the blessing Christ, and in the corners, the four seasons, represented as anthropomorphic figures, match with the corresponding zodiac signs.^63^ Each sign is linked to a specific part of the human body, resulting in a combination called *melothesia* that varies according to different cultures and authors. According to this doctrine, when the sun entered the sign of Aries, people were more prone to suffer from ailments to the head, while when it entered the sign of Taurus, they should be careful about ailments to the neck, and so on.^64^ The *rota* is followed by information about the position of the sun throughout the year and by an explanation of the zodiac signs according to meteorological events and Greek mythology. For instance, Taurus is linked to the myth of Zeus and Europe, while Gemini is traced back to the twins Castor and Pollux. Libra, instead, stands for the equal length of day and night on the autumn equinox, while Aquarius and Pisces are explained by the abundance of rain in the winter months.^65^

**Fig. 2:**
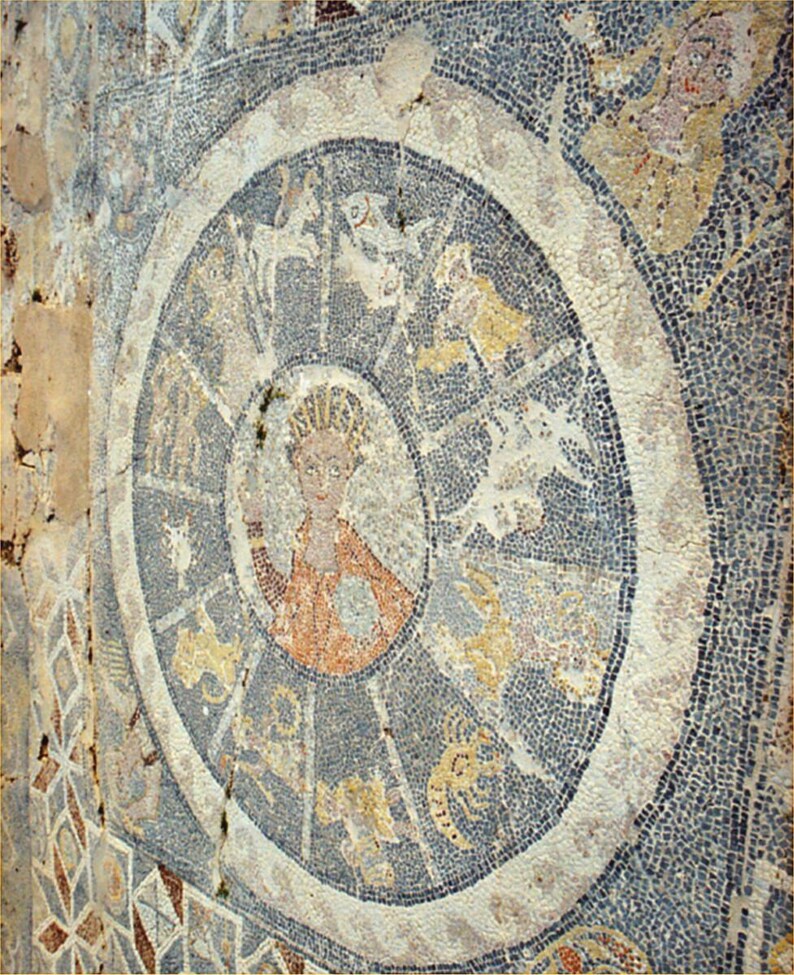
Fifth-century mosaic in the Tallaras Baths, on Astypalaia Island, Greece. Source: Wikimedia Commons. Accessed on 15 July 2024. Public domain.

**Fig. 3:**
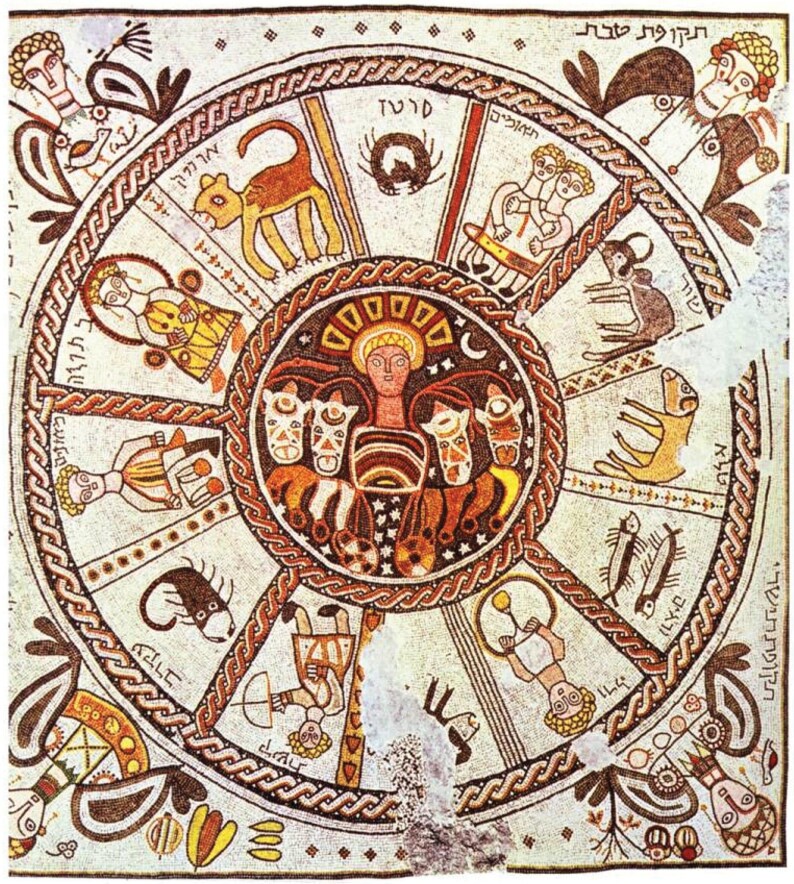
Sixth-century mosaic in the Beith Alpha synagogue, Heftziba, Israel. Source: Wikimedia Commons. Accessed on 15 July 2024. Public domain.

While Ernest Wickersheimer and Harry Bober identified this astrological diagram as the earliest representation of zodiacal medical astrology in Latin Europe, this iconographical model had a longer tradition in the Mediterranean area.^66^ Despite attributing the production of the images to a *scriptorium* in central Italy, Giuseppa Zanichelli detected Byzantine influences in the iconography of the *rota*, suggesting the persistence of eastern stylistic models in early medieval Italy, or even a Byzantine direct source of inspiration.^67^ In the line of Zanichelli’s intuition, an original comparison of this *rota* to late antique archaeological evidence supports the existence of a shared iconographical model of the zodiac that circulated at least since the first century CE in the Mediterranean area. Identical mosaic examples with the sun in the middle, the zodiac signs orbiting around it in circle, and the seasons in the four corners, have been found in first-century CE Palmyra, second-century Antioch, in late antique Sparta and on the island of Astypalaia (Figure 2), and in half a dozen fourth- to sixth-century synagogues in ancient Palestine (Figure 3).^68^ This iconographical model of the zodiac appears again in the Vatican manuscript Vat.gr. 1291, a Greek copy of Ptolemy’s *Handy Table*, tentatively dated to the ninth century.^69^ While it is currently impossible to argue whether the illuminator, possibly Philagathos himself, observed one of these mosaics while travelling to Byzantium, or if he drew inspiration from pre-existing manuscript sources, the *rota* in codex Parisinus is clearly a remnant of the classical past that persisted in its ancient iconography up to the early Middle Ages but would soon be reshaped in the late medieval diagram of the Zodiac Man, or *homo signorum*.

In contrast to the late medieval and early modern popularity of *melothesia*, our compiler explicitly expressed his distrust in this doctrine by describing the *rota* as the ramblings of philosophers (*philosophorum deliramenta*), either rejecting the micro-macrocosmic correspondence or the astrological influence on the body. Yet, he actively included the information in his medical compendium. While offering the chance to explain the Greek origins of the zodiac signs, the compiler’s rejection of astrological influence was also functional to the introduction of a different interest in astronomical movements, which helped to understand the passing of seasons and the consequent changing of winds, namely the evident causes of health and disease. To this purpose, Johannes noted the length of the solar year and its periodisation in 12 segments, respectively associated with a zodiac sign. He conveyed the same kind of information about the lunar month, but he did not suggest any astrological influence, which was predominant in other texts called *lunaria*.^70^

**Fig. 4:**
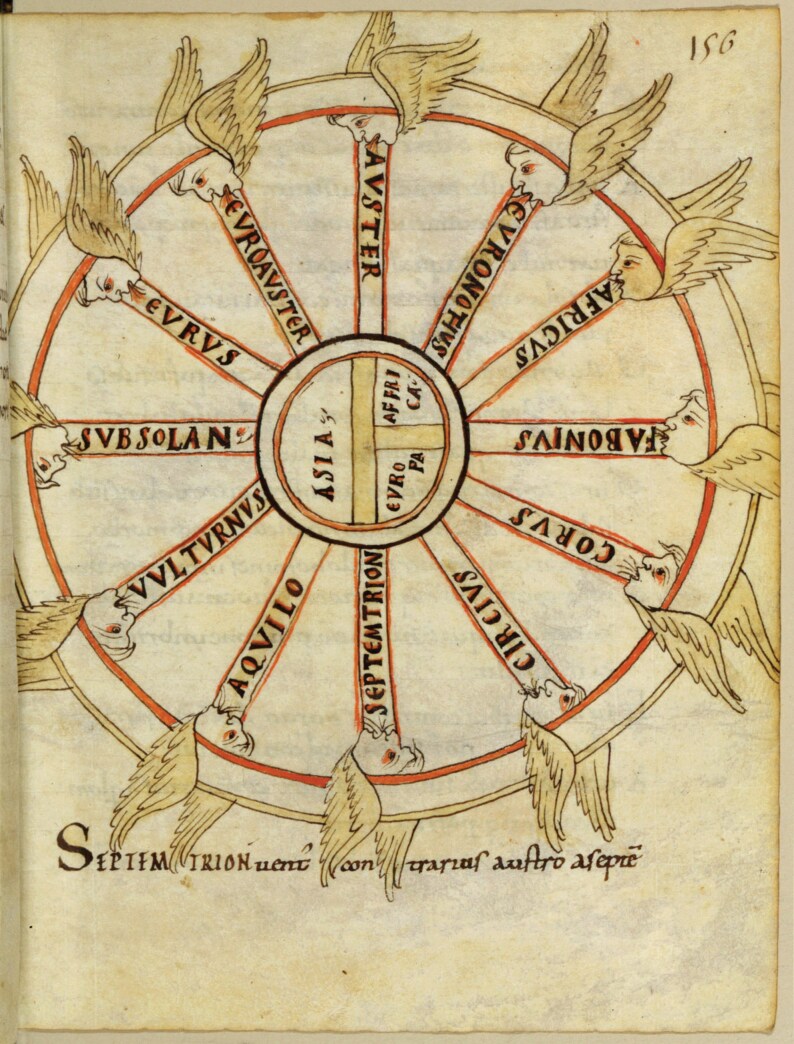
*Rota* with the winds. Ms Paris, BnF, Lat. 7028, 156r. Courtesy of the Bibliothèque nationale de France.

The observation of celestial movements proved to be fundamental to the second *rota*, where winds became more prominent explanatory tools. In Figure 4, 12 European winds are represented by winged faces that blow in the direction of the wind. In the line of the traditional representational system designed by Aristotle, winds are placed according to their origin on the circle of the horizon, which is here clarified by a T-O map in the inner circle.^71^ As Barbara Obrist has argued, such images with personifications of the winds were re-elaborations of earlier diagrammatic representations, which suggests that the wind *rota* in codex Parisinus responded to recent visual innovations, whereas the zodiacal *rota* was a product of classical antiquity. At the same time, Obrist noted that the short text that follows the image, called *On the virtues and natures of winds*, retained Peripatetic features in the diametrically opposite disposition of winds.^72^ After this short summary, Johannes reworked the sections on winds from the writings of Isidore of Seville, describing each wind according to the four primary Galenic qualities, and holding them responsible for changes in the temperature and the weather.^73^

Winds also appear in other sections of the manuscript. As explained in the reworking of two late antique texts resulting in the so-called *Liber Anathegore de pulsis et urinis*, winds and the weather influenced bodily humours and were responsible for their imbalances.^74^ In this way, the original authors implicitly explained aetiology with intrinsic causes, such as the corruption of the body or the spirit, and extrinsic causes related to food, drink, and especially corrupted air and noxious winds.^75^ In codex Parisinus, some excerpts from Celsus’ *De medicina* also described the effects of northern and southern, dry and moist winds, explaining that health depended on daily winds but also on the weather and the season in general.^76^ All these passages concerning the winds unveil the net of internal references and the cohesiveness of contents modelled on Celsian therapeutics.

## Ancient recipes and new simples

The compiler’s interests in medical epistemology, Greek vocabulary, regimen, surgery, and the winds are complemented by practice-led scribal activities concerning the preparation of medications. Experimental and learned recipes appear intertwined in Johannes’ medical activity.^77^ The aforementioned marginal recipe for coughing in manuscript Vat.lat. 5951 is linked to the authority of Johannes Philagathos, arguably its inventor, and it is described as *probata*, a widely used adjective for tested recipes. The multiple sets of recipes in Parisinus, instead, are codified authoritative formulas that came from pre-existing *antidotaria*. Almost none of them relied on the validation of users’ experience but rather on the name of their prestigious, and often legendary, inventor, whether it was Galen or Julius Caesar. To the pharmaceutical extracts from Celsus’ *De medicina*, Johannes added anonymous recipes for ointments and medicinal oils, and in the gynaecological section of the manuscript, he included recipes for emmenagogues and a series of treatments for the suffocation of the womb from the *Liber de muliebria*.^78^ Two recipes for emmenagogues, defined with both the Greek *emagogon* and the Latin *emplastrum sanguinarium*, recommended the use of pessaries to insert in the vaginal canal, which is here referred to as ‘fisin’, from the Greek φύσις (‘nature, origin, birth’), betraying the Greek origins of the recipes and an imperfect process of translation.^79^

A substantial part of our compiler’s pharmacological account concerns ancient medicinal recipes that likely depended on Byzantine areas as their primary site of transmission, and which display long-distance commercial relationships with the eastern Mediterranean. While recent studies have uncovered the role of Byzantine writers in the systematic reorganisation of ancient Greek pharmaceutical knowledge extracted from Dioscorides and Galen, Byzantine culture also played an important role in the European reception of the Arabic pharmacopeia, which expanded the traditional Greek pharmacy with imports from Central Asia, India, and the Indonesian archipelago.^80^ In southern Europe, where the Byzantine influence was stronger, the nomenclature of *materia medica* was rich and precise, and it is therefore presumable that Italian markets offered an ample choice of simples. Due to their long journey and limited availability, these products were expensive, and yet, monks often made use of them, revealing the economic wealth of monasteries like Nonantola.^81^

In addition to recipes, Johannes provided further information about medicinal ingredients by copying a likely pre-existent but virtually unknown list of more than one hundred simples divided into eight classes (pigments, metals, resins, plants, roots, flowers, seeds, and biles).^82^ These simples show already well-established commercial connections to the Islamicate worlds in Spain and North Africa and the Far East. Oriental simples like nutmeg and cinnabar were first described in Europe in the eighth century, when the substances themselves and information about them reached Frankish centres of manuscript production, arguably passing through the Italian peninsula and slowly being assimilated into western recipes through personal witness, oral communication, and commercial exchanges.^83^ Never mentioned in ancient medicine, zedoary, galangal, nutmeg, and *anacardium* appear in this list, recorded among the pigments.^84^ Perfumed substances, such as musk and ambergris, respectively coming from the mountains of Central Asia and from the shores of the Indian Ocean, are also listed here.^85^ Other exotic ingredients like *cozumber* and *confita*, which probably consist of by-products of storax, are here listed as resins, but they also appear as ingredients in the recipes for ointments, demonstrating the gradual inclusion of exotic simples in the European pharmacopeia.^86^

Importantly, medieval users revised ancient recipes by incorporating new ingredients. The section about ointments includes a recipe for a powerful sweet and gummy preparation (*electuarium*) called ‘diarodes Iulii’. This compound was traditionally named after its main ingredient, the rose, from the Greek ῥόδον (*rhodon*), and this particular version was attributed to Julius Caesar.^87^ This medication treated many conditions of the stomach and the lungs, colds, dizziness, and diarrhoea, and included over seventy exotic ingredients, such as two kinds of pepper, cinnamon, ginger, cardamom, zedoary, amber, and balsam. Semi-precious ingredients like pearls and red coral are also employed, suggesting that the compound was intended for wealthy users. Roses, however, did not appear as ingredients in this version of the recipe, suggesting its evolution throughout the centuries and, at the same time, the permanence of its name and attribution to Caesar, whose authority guaranteed the compound’s efficacy.^88^ Predating of a century the Salernitan collections of recipes known as *Antidotarium magnum* and *Antidotarium Nicolai*, which historians still need to subject to systematic comparison with earlier and contemporary manuscripts, codex Parisinus provides evidence of the enduring circulation of ancient recipes and the assimilation of eastern simples in the final decades of the Byzantine hegemony in southern Italy, challenging the primacy of Salerno and showing that the renewal of ancient medicine was an intermittent, yet diffused process of innovation.

## Healing beyond medicine

While the contents of codex Parisinus described so far have followed a consistent Celsian approach to medicine, addressing regimen, *materia medica*, and surgery as preferred therapeutical strategies oriented towards evident causes of disease, two short sections of the manuscript complicate the picture, providing insights into the eclectic nature of the compiler’s approach to the multifaceted world of healing. After discussing pharmacological remedies, the compiler offered treatments that involved stones described in Isidore’s *Etymologies* and Damigeron’s *De virtutibus lapidum*, a Hellenistic Greek lapidary that was translated into Latin in late antiquity.^89^ By providing these alternatives to Celsian therapeutics, our compiler likely considered all these therapies as equally effective, but he left many unanswered questions about the occult properties of these stones.

In antiquity, Galen and Dioscorides established two different classes: stones that physicians administered grinded had explicable virtues connected to the items’ specific complexion; gems that stayed intact, instead, were considered amulets powered by unknown, occult properties.^90^ Our compiler embraced a syncretic approach. He considered some stones for their natural virtues, as some of them also appear in the list of simples as metals.^91^  *Lapis medium* was applied to the eyes; a poultice of pounded stone and breastmilk was considered to heal blindness, but washing someone’s face with a mixture of pounded stone and water supposedly could turn one blind. Pounded and mixed with water, *lapis galathitis* fostered the production of breastmilk, while *lapis hematites* healed snake bites.^92^

Other stones, instead, worked as amulets in medical and non-medical matters. The most evident example is the stone called magnet, which was especially noted for its occult qualities.^93^ In the excerpts from Damigeron’s lapidary, two kinds of magnet are described for their property to attract iron, but while the *lapis magnete* had purgative virtues and should be administered in water to people suffering from dropsy, the *magnes lapis indicus* had non-medical properties. Reportedly, thieves used it to rob houses; when placed over coals in the corners of a house, this stone would emit a lot of smoke and make the residents flee, fearing the house would collapse. This stone could also reveal a woman’s faithfulness to her husband when hidden under her pillow. If she had had sexual intercourse with another man, the stone would emit such a disgusting smell that she would fall off the bed.^94^ While the *lapis magnete* was administered grinded like many other drugs, the *magnes lapis indicus* was treated as an amulet but without magical qualities, as the text does not include Damigeron’s references to people in Colchis, who ascribed magical powers to this stone.

While the compiler trusted the powers of stones in a variety of different matters, another section of the manuscript discloses the compiler’s medical application of religious practices. In comparison to Carolingian manuscripts like the *Lorscher Arzneibuch*, which provided meticulous Christian apologies of medicine, the absence of religious justifications of medical practice and the persistence of Greek pagan elements in codex Parisinus suggest the compiler’s efforts at integrating ancient medicine with some elements of religion. Curiously, religion emerges explicitly only once, on a scrap leaf with a prayer that begs God and the archangel Raphael to bless the preparation of a medication and free humans from the bonds of illness and evil. Medieval medical prayers often invoked the intervention of Raphael, who revealed humans how to use the products of the creation, teaching them the art of medicine. In this prayer, the compiler recalled a Biblical episode from the book of Tobit, where Raphael taught Tobit how to use fish gall as a poultice to heal his father’s eyes, affected by white spots that turned him blind.^95^ The book of Tobit was hence a suitable source to explain the use of pharmaceutical remedies while maintaining the subordination of simples’ medicinal virtues to God’s ultimate salvific power.^96^

By reading the prayer out loud while preparing the medication, the healer could perform a ritual that belonged to an intertwined realm of medical and religious practices.^97^ The presence of this prayer in the manuscript does not question the compiler’s Rationalist orientation, with its attention to causal explanations of disease, but rather adds a further layer of meaning that enriches our understanding of early medieval healing practices. On the one hand, healing practices that involved the preparation of medications sought the involvement of divine powers to be successful. On the other hand, at least in Tobit’s case, the archangel taught him how to cure his father’s eyes with the art of medicine, using natural means. To the compiler, prayers and poultices arguably supported each other as parts of the same medical culture, which equally concerned powerful stones and noxious winds, ancient pagan myths, and instructions for cauterisation, questioning the linear teleology of the western history of medicine and offering a glimpse in one of the complex healing cultures of early medieval Europe.^98^

## Conclusion

Codex Parisinus offers a dynamic picture of early medieval medicine by showing the persistent permeability of classical, late antique, and eastern Mediterranean medicine before the rise of the School of Salerno. This tenth-century Greek-speaking writer-compiler, who lived between the Ottonian and the Byzantine Empires in overlooked cultural crossroads like Nonantola, relied on ancient sources, especially on Rationalist ideas of healing and visual representations of the heavens, while being receptive to recent developments coming from the Arabic and Byzantine worlds. The selection of texts in codex Parisinus, which complies to the therapeutical guidelines offered by Celsus’ *De medicina*, suggests a dense net of intellectual and commercial connections to the south-eastern Mediterranean, and this compilation shows that the results of cross-cultural transfers were received well before the development of major translation centres like Salerno and Toledo.^99^

While engaging with recent medical developments, codex Parisinus is also evidence of the stratification and enduring transmission of ideas and practices, and it engages with *longue durée* histories of practical medicine and epistemology. The emergence of the Rationalist sect through the rare witness of Celsus’ *De medicina*, and the persistence of Galenic classifications of drugs, reveal the compiler’s steady reliance on ancient medical epistemology and chains of causation in explaining disease, which had a pivotal role in establishing the primacy of Scholastic and learned medicine in the late Middle Ages and the early modern age. Furthermore, in spite of the compiler’s distrust in astrological influence, the presence of the zodiacal *rota* hints at the continuous transmission of ideas that originated in ancient Mesopotamia and persisted as meaningful ornamental features in late antique laic and religious sites in the eastern Mediterranean area. At the same time, the astrological *rota* is a harbinger of ideas that developed in western Europe a few centuries later, with the visual codification of *melothesia* in the epistemic diagram known as Zodiac Man. Similarly, codex Parisinus helps situate the millenary activity of making and collecting recipes in formal and informal contexts.^100^ The long-lasting circulation of multi-layered collections of recipes and their evolution with the introduction of new ingredients at the hands of late antique and medieval medical practitioners, show the malleability of this epistemic genre and reveal the agency of eclectic compilers like Johannes. In his medical activity, he borrowed from pre-existent *antidotaria*, while inventing new recipes extant only on the margins of other manuscripts.

The organic reading of codex Parisinus as a cohesive textual unit designed by one compiler over an unknown amount of time challenges previous understandings of early medieval medical miscellanies. When textual and material collections are described as miscellanies, the parameters of authorship, intentionality, and order often blur, as they imply a confused process of assemblage that brought together items that naturally would not belong with each other. While early modern efforts to systematise the natural world significantly affected the organisation of textual products like herbals and the disposition of objects in cabinets and museums, often juxtaposing purposes of amusement to actual scientific advancements, early medieval miscellanies resist being classified in similar ways.^101^ Forcing medical miscellanies into a rigid and tidy scheme hinders the possibility of acknowledging the diachronic evolution of the compilers’ interests and impairs a syncretic understanding of these intellectual products. The compiler of codex Parisinus merged ancient sources and newly circulating information to shape an intellectually coherent and cohesive medical account that, in its consistent but heterogeneous nature, resists the linearity of (early) modern rationality and its inherent teleological narratives. This new understanding of medical miscellanies demonstrates the agency of early medieval compilers and discloses myriads of dynamic assemblages of contents previously disregarded as the result of nonsensical intellectual processes and corrupted lines of transmission.

## Footnotes

1 Augusto Beccaria, *I codici*  *di medicina del periodo presalernitano* (Rome: Edizioni di storia e letteratura, 1956), 152–6. Ernest Wickersheimer, *Les manuscrits latins de médecine du haut Moyen Age dans les bibliothèques de France* (Paris: Éditions du CNRS, 1966), 84–87. See also the recently improved description in the BnF catalogue. URL: < https://archivesetmanuscrits.bnf.fr/ark:/12148/cc661962>, accessed on 22 July 2024.

2 For descriptions of early medieval medical manuscripts, see Beccaria*, I codici*, and Wickersheimer, *Les manuscrits*. See also Florence Eliza Glaze, ‘The Perforated Wall: The Ownership and Circulation of Medical Books in Medieval Europe, ca. 800-1200’ (unpublished PhD thesis, Duke University, 1999). Klaus-Dietrich Fischer, ‘Dr Monk’s Medical Digest’, *Social History of Medicine*, 2000, 13, 239–51. Clare Pilsworth, *Healthcare in Early Medieval Northern Italy* (Turnhout: Brepols, 2014), esp. 78–93. Margaret Connolly, ‘Practical Reading for Body and Soul in Some Later Medieval Manuscript Miscellanies’, *Journal of the Early Book Society*, 2017, 10, 151–74.

3 John Grattan and Charles Singer, *Anglo-Saxon Magic and Medicine* (London: Oxford University Press, 1952), 16, 24. Gerhard Baader, ‘Early Medieval Latin Adaptations of Byzantine Medicine in Western Europe’, *Dumbarton Oaks Papers*, 1984, 38, 251–9. Anne van Arsdall, ‘Reading Medieval Medical Texts with an Open Mind’, in Elizabeth Lane Furdell, ed, *Textual Healing: Essays on Medieval and Early Modern Medicine* (Leiden: Brill, 2005), 9–29.

4 Faith Wallis, ‘The Experience of the Book: Manuscripts, Texts and the Role of Epistemology in Early Medieval Medicine’, in Don Bates, ed, *Knowledge and the Scholarly Medical Traditions* (Cambridge: Cambridge University Press, 1995), 101–26.

5 Peregrine Horden, ‘What’s Wrong with Early Medieval Medicine?’, *Social History of Medicine*, 2009, 24, 5–25.

6 Marilena Maniaci, ‘The Medieval Codex as a Complex Container: The Greek and Latin Traditions’, in Michael Friedrich and Cosima Schwarke, eds, *One-Volume Libraries: Composite and Multiple-Text Manuscripts* (Berlin: De Gruyter, 2016), 27–46.

7 Anna Dorofeeva, ‘Visualizing Codicologically and Textually Complex Manuscripts’, *Manuscript Studies*, 2019, 4, 334–60, and ‘Reading Early Medieval Miscellanies’, in Barbara Shailor *et al.*, eds, *Scribes and the Presentation of Texts* (Turnhout: Brepols, 2021), 495–514. Alessandro Bausi, Michael Friedrich, and Marilena Maniaci, eds, *The Emergence of Multiple-Text Manuscripts* (Berlin: De Gruyter, 2019). Michael McVaugh, ‘A Miscellany? Or the Evolution of a Mind? Ms Munich CLM 534’, in Agostino Paravicini Bagliani, ed, *Les miscellanées scientifiques au Moyen Âge* (Florence: SISMEL, Edizioni del Galluzzo, 2019), 71–86.

8 On ancient medical sects, see Philip van der Eijk, ed, *Ancient Histories of Medicine: Essays in Medical Doxography and Historiography in Classical Antiquity* (Leiden: Brill, 1999).

9 For late medieval Rationalism, see Roger French, *Medicine before Science. The Business of Medicine from the Middle Ages to the Enlightenment* (Cambridge: Cambridge University Press, 2003), 88–97, and Michael McVaugh, *The Rational Surgery of the Middle Ages* (Florence: SISMEL, Edizioni del Galluzzo, 2006). For early modern reappraisals of ancient medical epistemology, see, for instance, Craig Martin, ‘Galenic Causation in the Practical and Theoretical Medicine of Giambattista da Monte’, in Matteo Favaretti Camposampiero and Emanuela Scribano, eds, *Galen and the Early Moderns* (Cham: Springer, 2022), 37–53.

10 Wallis, ‘The Experience’. Horden, ‘What’s Wrong’. James Palmer, ‘Merovingian Medicine between Practical Art and Philosophy’, *Traditio*, 2023, 78, 17–45.

11 Loren MacKinney, ‘Tenth-Century Medicine as Seen in the *Historia* of Richer of Rheims’, *Bulletin of the Institute of the History of Medicine*, 1934, 2, 347–75. Pilsworth, *Healthcare*, 209–15. Meg Leja, *Embodying the Soul. Medicine and Religion in Carolingian Europe* (Philadelphia, PA: University of Pennsylvania Press, 2022), 103–64.

12 Danielle Jacquart and Charles Burnett, eds, *Constantine the African and ‘Alī ibn al-‘Abbās al-Ma**ǧ**ūsī**: The* Pantegni *and Related Texts* (Leiden: Brill, 1994). Francis Newton, *The Scriptorium and Library at Monte Cassino, 1058-1105* (Cambridge: Cambridge University Press, 1998). Charles Burnett, *Arabic into Latin in the Middle Ages*. *The Translators and their Intellectual and Social Context* (Farnham: Ashgate, 2009). Erik Kwakkel and Francis Newton, *Medicine at Monte Cassino: Constantine the African and the Oldest Manuscript of his* Pantegni (Turnhout: Brepols, 2019).

13 Charles Haskins, *The Renaissance of the Twelfth Century* (Cambridge, MA: Harvard University Press, 1927). Charles Burnett, ‘The Twelfth-Century Renaissance’, in David Lindberg and Michael Shank, eds, *The Cambridge History of Science* (Cambridge: Cambridge University Press, 2013), II, 365–84.

14 Innocenzo Mazzini and Nicoletta Palmieri, ‘L’École médicale de Ravenne: Programmes et méthodes d’enseignement, langue, hommes’, in Philippe Mudry and Jackie Pigeaud, eds, *Les écoles médicales à Rome* (Geneva: Librairie Droz, 1991), 285–310. Florence Eliza Glaze, ‘Master-Student Medical Dialogues: The Evidence of London, British Library, Sloane 2839’, in Patrizia Lendinara, Loredana Lazzari, and Maria Amalia D’Aronco, eds, *Form and Content of Instruction in Anglo-Saxon England in Light of Contemporary Manuscript Evidence* (Turnhout: Brepols, 2007), 20–23. On Salerno, see Paul Oskar Kristeller, ‘The School of Salerno: Its Development and Its Contribution to the History of Learning’, *Bulletin of the History of Medicine*, 1945, 17, 138–94, and Monica Green, *The Trotula. A Medieval Compendium of Women’s Medicine* (Philadelphia, PA: University of Pennsylvania Press, 2001), 3–14.

15 Lennart Lehmhaus and Matteo Martelli, eds, *Collecting Recipes: Byzantine and Jewish Pharmacology in Dialogue* (Berlin: De Gruyter, 2017). Petros Bouras-Vallianatos, ‘Galen in Byzantine Medical Literature’, in Petros Bouras-Vallianatos and Barbara Zipser, eds, *Brill’s Companion to the Reception of Galen* (Leiden: Brill, 2019), 86–110, and ‘Cross-Cultural Transfer of Medical Knowledge in the Medieval Mediterranean: The Introduction and Dissemination of Sugar-Based Potions from the Islamic World to Byzantium’, *Speculum*, 2021, 96, 963–1008. Panagiotis Athasanopulos, ed, *Translation Activity in Late Byzantine World* (Berlin: De Gruyter, 2022). Petros Bouras-Vallianatos and Dionysios Stathakopoulos, eds, *Drugs in the Medieval Mediterranean. Transmission and Circulation of Pharmacological Knowledge* (Cambridge: Cambridge University Press, 2023).

16 Giuseppe Billanovich and Mirella Ferrari, ‘La trasmissione dei testi nell’Italia nord-occidentale’, in *La cultura antica nell’Occidente latino dal VII all’XI secolo* (Spoleto: CISAM, 1975), 334–37. Sébastien Bricout, ‘La connaissance du *De medicina* de Celse au tournant du Xe siècle’, *Revue d’histoire des textes*, 2009, 4, 289–98.

17 Ms Parisinus, 133v: ‘Litoris ad portum lector hoc opus aspice nostrum/ et multis plenam pigmentis sume sagenam/ quam scriptor scripsi sacer Iohannes atque refixi/ viribus adiunctis iussum sectatus alumpni/ correxi partem summo conamine libri/ feci quod potui, quod non invitus omisi’.

18 Ovid, *Amores* III.11b.3. Friedrich Marx, *Cornelii Celsi quae extant* (Leipzig: Teubner, 1915), XXXVII.

19 Bricout, ‘La connaissance’. For a complete overview of Philagathos’ life, see Wolfgang Huschner, ‘Giovanni XVI, antipapa’, in *Enciclopedia dei Papi* (Rome: Istituto della Enciclopedia Italiana, 2000), II, 112–16.

20 Thietmar of Merseburg, *Chronicon* IV.30, in MGH, *SS rer. Germ. N. S.* 9, 167. John Deacon, ‘Cronaca veneziana’, in Giovanni Monticolo, ed, *Cronache veneziane antichissime* (Rome: Istituto storico italiano, 1890), 153–4. *Die Briefsammlung Gerberts von Reims*, in MGH, *Briefe d. dt. Kaiserzeit* 2, 261. On Rossano’s importance as a centre of Greek language and culture, and on the influential figure of Saint Nilus, also born in Rossano and relevant to the last part of Philagathos’ life, see the contributions of Anna Maria Ieraci Bio, ‘Notazioni mediche nella *Vita Nili*’, and Santo Lucà, ‘Attività scrittoria e culturale a Rossano: da San Nilo a S. Bartolomeo da Simeri (secoli X-XII)’, in *Atti del Congresso Internazionale su S. Nilo di Rossano* (Grottaferrata: Scuola tipografica italo-orientale S. Nilo, 1989), 25–73 and 441–61. See also Salvatore Cosentino, ed, *A Companion to Byzantine Italy* (Leiden: Brill, 2021), 751.

21 Phyllis Jestice, *Imperial Ladies of the Ottonian Dynasty. Women and Rule in Tenth-Century Germany* (Cham: Palgrave Macmillan, 2018), 237, 244.

22 See the contributions of Karl Leyser, Rosamond McKitterick, and Jacqueline Lafontaine-Dosogne in Adelbert Davids, ed, *The Empress Theophano: Byzantium and the West at the Turn of the First Millennium* (Cambridge: Cambridge University Press, 1995). Rosamond McKitterick, ‘Ottonian Intellectual Culture in the Tenth Century and the Role of Theophanu’, *Early Medieval Europe*, 1993, 2, 53–74. Judith Herrin, *Unrivalled Influence: Women and Empire in Byzantium* (Princeton, NJ: Princeton University Press, 2013), 238–60.

23 Walter Berschin, *Greek Letters and the Latin Middle Ages*, J. Frakes (trans) (Washington, DC: Catholic University of America Press, 1988), 172–85. See also Agostino Pertusi, ‘Bisanzio e l’irradiazione della sua civiltà in Occidente nell’alto medioevo’, in *Centri e vie di irradiazione della civiltà nell’alto Medioevo* (Spoleto: CISAM, 1964), 101–6. Karl Leyser, ‘The Tenth Century in Byzantine-Western Relationships’, in Derek Baker, ed, *Relations between East and West in the Middle Ages* (Edinburgh: Edinburgh University Press, 1973), 43–46. André Gillou, *Culture et société en Italie byzantine (VIe-XIe siècle)* (London: Variorum, 1978), 128. Vera von Falkenhausen, ‘Between Two Empires: Southern Italy in the Reign of Basil II’, in Paul Magdalino, ed, *Byzantium in the Year 1000* (Leiden: Brill, 2003), 135–59. Birger Munk Olsen, *L’étude des auteurs classiques latins aux XIe et XIIe siècles* (Paris: CNR Éditions, 2014), IV.2, 135, 396–97.

24 This inventory is extant in the famous *Lorscher Arzneibuch*, namely, manuscript Bamberg, Staatsbibliothek, Msc.Med. 1, 42v: ‘Isti sunt libri tercii imperatoris Ottonis, quos Iohan Placentiae invenit sibi servatos: duos libros Orosii, Persium, duos libros Titi Livii non minimam partem, medicinalem unum, duos capitulares, Fulgentium unum simul cum Ortographia Isidori episcopi, in Isagogas Porphyrii minus commentum Boecii, duos Glossarios’. Florentine Mütherich, ‘The Library of Otto III’, *Bibliographia*, 1986, 4, 16–17.

25 Karl and Matilde Uhlirz, *Jahrbücher des deutschen Reiches unter Otto II und Otto III* (Berlin: Duncker&Humblot, 1902), 406 n73. Mütherich, ‘The Library’, 11–25. See also Alfried Wieczorek and Hans-Martin Hinz, *Europe’s Centre Around AD 1000* (Stuttgart: Theiss, 2000), I, 145–60.

26 MGH, DD O II, 330: ‘Posthac consultu sapientium reduxi oculos meos ad aulicos, inter quos quondam archimandritem et consecretalem meum Iohannem nomine repperi probis moribus ornatum, pudicum, sobrium, docibilem, Greca sciencia non ineruditum tociusque prudencie et sanctitatis fulgore preclarum’. For a definition of archimandrite, see Alexander Kazhdan, *The Oxford Dictionary of Byzantium* (Oxford: Oxford University Press, 1991), I, 156.

27 According to Bricout, codex Parisinus was produced in Nonantola in the 980s, and between 989 and 997, it was in Reims, where Gerbert of Aurillac and possibly Richer of Reims read it. Bricout, ‘La connaissance’.

28 Ms Vat.lat. 5951, 124r: ‘Sacerdos dei Silvester ora pro nobis, ut tuis semper precibus piis protege’. Billanovich and Ferrari, ‘La trasmissione’, 332–38. Other scholars questioned Billanovich’s dating of codex Parisinus, claiming that it was produced in southern Italy in the mid-eleventh century. François Avril and Yolanta Zaluska, *Manuscrits enluminés d’origine italienne* (Paris: Bibliothèque nationale de France, 1980), I, 27–28. Paola Supino Martini, *Roma e l’area grafica romanesca, secoli X-XII* (Alessandria: Edizioni dell’Orso, 1987), 40 n39, 167. The attribution of manuscript V to Nonantola is also disputed. A palaeographical exam of the handwriting and musical notation led Branchi to conclude that the codex belonged to northern Italy. Mariapia Branchi, *Lo scriptorium e la biblioteca di Nonantola* (Nonantola: Centro studi storici nonantolani, 2011), 154–55. Friedrich Marx, a twentieth-century editor of *De medicina*, linked the Celsian excerpts found in Parisinus to the text in manuscript V, but he could not provide conclusive philological evidence to establish a direct filiation of Parisinus from manuscript V because of the compiler’s invasive textual emendations and manipulation of contents. Marx, *Celsi*, XXXIV–XXXV.

29 Beccaria, *I codici*, 312–13. Munk Olsen, *L’étude*, I, 92–93. Claire Burridge, *Carolingian Medical Knowledge and Practice, c.775-900* (Leiden: Brill, 2024), 302.

30 Ms Vat.lat. 5951, 66v: ‘Ad incensum. Recipit sinonum piper ana ⋮.II. siler zinziber cinnamomum euforbium eruce semina maratri opobalsamum ana ⋮.S. satureia sansucum enule radix ana ⋮.I. mel.’, and 68v: ‘Cataputium ad tussi probata et disentericis Iohannis Calabri. Recipit opium castoreum galbanum storacem mirra piper equaliter cum vino collige et fac cataputium in modum ciceris maioris et da in nocte .III. aut .V’. Another recipe for incense appears in the same manuscript on 1v, but Claire Burridge dated it to the ninth century. Burridge, *Carolingian Medical Knowledge*, 302, 355.

31 The foundation of this Benedictine monastery dates to the mid-eighth century, when Lombard rulers fought for control over Italy, relying on major religious institutions to support their claims. In 752, the Lombard king Aistulf appointed his brother-in-law Anselm abbot of the newly founded community in Nonantola, a small town near Modena that controlled the roads connecting Bologna to Piacenza and Verona. Karl Schmidt, ‘Anselm von Nonantola. *Olim dux militum—nunc dux monachorum*’, *Quellen und Forschungen aus italienischen Archiven und Bibliotheken,* 1967, 47, 1–122. Maria Stella Zoboli, *Il Monastero di San Silvestro di Nonantola all’epoca dell’abbaziato di Pietro (804-824/25)* (Nonantola: Centro di studi storici nonantolani, 1997). See also Nicholas Everett, *Literacy in Lombard Italy, c. 568-774* (Cambridge: Cambridge University Press, 2003).

32 Paolo Peri, ‘Antiche reliquie tessili dell’Abbazia di Nonantola’, in Riccardo Fangarezzi, Paolo Golinelli, and Alba Maria Orselli, eds, *Sant’Anselmo di Nonantola e i santi fondatori nella tradizione monastica* (Rome: Viella, 2006), 239–60. Alessandra Cianciosi, Cecilia Moine, and Lara Sabbionesi, ‘Tavola e *scriptorium*: cultura materiale nel monastero di S. Silvestro a Nonantola’, in Marialuisa Bottazzi *et al.*, eds, *La società monastica nei secoli VI-XII: sentieri di ricerca* (Trieste: CERM, 2016), 428–29. Chris Wickham, *The Donkey and the Boat: Reinterpreting the Mediterranean Economy, 950-1180* (Oxford: Oxford University Press, 2023), 528.

33 Bernard Hamilton and Patricia McNulty, ‘*Orientale lumen et magistra Latinitas*: Greek Influences on Western Monasticism (900-1100)’, in Bernard Hamilton, ed, *Monastic Reform, Catharism and the Crusades (900-1300)* (London: Variorum, 1979), 198. Franco Dell’Aquila, ‘Matera e Nonantola’, *Mathera*, 2019, 8, 141–45. See also Steven Vanderputten, *Medieval Monasticisms: Forms and Experiences of the Monastic Life in the Latin West* (Berlin: De Gruyter, 2020), 68.

34 Giuseppe Gullotta, *Gli antichi cataloghi e codici della abbazia di Nonantola* (Vatican City: Biblioteca Apostolica Vaticana, 1955). José Ruysschaert, *Les manuscrits de l’abbaye de Nonantola: table de concordance annotée et index des manuscrits* (Vatican City: Biblioteca Apostolica Vaticana, 1955). Bernhard Bischoff, ‘Manoscritti nonantolani dispersi di epoca carolingia’, *Bibliofilia*, 1983, 85, 99–124. Massimiliano Bassetti, ‘Scrivere leggere conservare nelle grandi abbazie (750-1100)’, in *Monachesimi d’Oriente e d’Occidente nell’alto Medioevo* (Spoleto: CISAM, 2017), 1246–52.

35 Giorgio Cencetti, ‘*Scriptoria* e scritture nel monachesimo benedettino’, in *Il monachesimo nell’alto Medioevo e la formazione della civiltà occidentale* (Spoleto: CISAM, 1957), 202–04. Marco Palma, ‘Nonantola e il Sud. Contributo alla storia della scrittura libraria nell’Italia dell’ottavo secolo’, *Scrittura e civiltà*, 1979, 3, 77–88, and ‘Alle origini del “tipo di Nonantola”: nuove testimonianze meridionali’, *Scrittura e civiltà*, 1983, 7, 141–9. Mirella Morelli and Marco Palma, ‘Indagine su alcuni aspetti materiali della produzione libraria a Nonantola nel secolo IX’, *Scrittura e civiltà*, 1982, 6, 23–98. Maria Pia Branchi, ‘Le origini della miniatura nonantolana’, in Giovanni Spinelli, ed, *Il monachesimo italiano dall’età longobarda all’età ottoniana (secc. VIII-X)* (Cesena: Badia di Santa Maria del Monte, 2006), 615–23. See also Ave Moderini, *La notazione neumatica di Nonantola* (Cremona: Athenaeum Cremonense, 1970).

36 Uwe Ludwig, ‘I *Libri memoriales* e i rapporti di fratellanza tra i monasteri alemanni e i monasteri italiani nell’alto Medioevo’, in Spinelli, ed, *Il monachesimo italiano*, 149–51. Richard Pollard, ‘*Libri di scuola spirituale*: Manuscripts and *Marginalia* at the Monastery of Nonantola’, in Lucio Del Corso and Oronzo Pecere, eds, *Libri di scuola e pratiche didattiche dall’Antichità al Rinascimento* (Cassino: Università di Cassino, 2010), 331–408, and ‘Nonantola&Reichenau. A New Manuscript of Heito’s *Visio Wettini* and the Foundations for a New Critical Edition’, *Revue bénédictine*, 2010, 120, 243–94.

37 Nicolas Drocourt, ‘Ambassades latines et musulmanes à Byzance: une situation contrastée’, *Byzantion*, 2004, 74, 354–58, and ‘Ambassadors as Informants and Cultural Brokers between Byzantium and the West’, in Falko Daim, Dominik Heher, and Claudia Rapp, eds, *Menschen, Bilder, Sprache, Dinge. Wege der Kommunikation zwischen Byzanz und dem Westen* (Mainz: Verlag des Römisch-Germanischen Zentralmuseums, 2018), 82.

38
*Annales Quedlinburgenses*, in MGH, *SS rer. Germ.* 72, 496–98. Louis Duchesne, ed, *Le Liber Pontificalis* (Paris: Thorin, 1892), II, 262. Eleanor Shipley Duckett, *Death and Life in the Tenth Century* (Ann Arbor, MI: University of Michigan Press, 1988), 124–28. Gerd Aldhoff, *Otto III*, P. Jestice (trans) (University Park, PA: Pennsylvania State University Press, 2003), 72–81.

39 Leja, *Healing the Soul*, 104–32.

40 Owsei Temkin, ‘Celsus’ “On Medicine” and the Ancient Medical Sects’, *Bulletin of the Institute of the History of Medicine*, 1935, 3, 249–64.

41 An incomplete edition of the collection is found in Rudolf Laux, ‘*Ars medicinae*. Ein frühmittelalterliches Kompendium der Medizin’, *Kyklos*, 1930, 3, 417–34. The text was edited again in Luigi Firpo, *Medicina medievale* (Turin: UTET, 1972), 24–35, but it lacks a critical apparatus. See also Jacques Jouanna, ‘Hippocrate et la Collection hippocratique dans l’*Ars medicinae*’, *Revue d’histoire des textes*, 1993, 23, 95–111. On Hippocratic biographies and deontological texts in the Middle Ages, see Ernst Hirschfeld, ‘Deontologische Texte des frühen Mittelalters’, *Archiv für Geschichte der Medizin*, 1928, 20, 364–71. Loren McKinney, ‘Medical Ethics and Etiquette in the Early Middle Ages: The Persistence of Hippocratic Ideals’, *Bulletin of the History of Medicine*, 1952, 6, 1–31. Pearl Kibre, ‘Hippocrates Latinus: Repertorium of Hippocratic Writings in the Latin Middle Ages III’, *Traditio*, 1977, 33, 280–82. Guy Sabbah, Pierre-Paul Corsetti, and Klaus-Dietrich Fischer, eds, *Bibliographie des textes médicaux latins. Antiquité et Haut Moyen Âge* (Saint-Étienne: Université de Saint-Étienne, 1987), 41–42. Klaus-Dietrich Fischer, ‘The “Isagoge” of Pseudo-Soranus: An Analysis of the Contents of a Medieval Introduction to the Art of Medicine’, *Medizinhistorisches Journal*, 2000, 35, 17–20.

42 Ms Parisinus, 1r-1v. Euripides, *Alcestis* 3–4.

43 Ms Parisinus, 1v: ‘Obum autem habet propter hoc ne sine transitu esset artificium, granata autem ostendit multos mores in hominibus per naturam, fialam autem propter multas petitiones laborantium. Hec ergo sic habetur in odiis capitaneis ut fabule dicunt’. This iconography of Asclepius and Hygeia was very popular in ancient Greece, as described by the second-century geographer Pausanias. Emma and Ludwig Edelstein, *Asclepius: A Collection and Interpretation of Testimonies* (Baltimore, MD: The Johns Hopkins Press, 1945), I, 347–8, 353–4; II, 87–90, 216.

44 Charles Du Cange *et al.*, *Glossarium mediae et infimae latinitatis* (Paris: Didot, 1845), IV, 695c, *oda*2.

45 Jody Pinault, ‘How Hippocrates Cured the Plague’, *Journal of the History of Medicine and Allied Sciences*, 1985, 41, 52–75, and *Hippocratic Lives and Legends* (Leiden: Brill, 1992), 36–60.

46 Ms Parisinus, 3v-4r. The complete list of Rationalist, Empiric, and Methodist physicians is edited in Manuela Tecusan, *The Fragments of the Methodists* (Leiden: Brill, 2003), I, 130–33. A comparison of this section in Parisinus to similar texts in manuscripts Bamberg, Staastbibliothek, Msc.Med. 1, 6r and St Gallen, Stiftsbibliothek, Cod.Sang. 751, 365 shows that these three versions belonged to structurally identical compilations of deontological, historical and physiological texts, suggesting a shared archetype for all these manuscripts. Codex Parisinus, however, is the only version where the names of Empiric and Methodist physicians are excised. For other early medieval texts on ancient medical sects, see Glaze, ‘Master-Student Medical Dialogues’, 11–29.

47 Temkin, ‘Celsus’ “On Medicine”’. Umberto Capitani, ‘A.C. Celso e la terminologia tecnica greca’, *Annali della Scuola Normale Superiore di Pisa*, 1975, 5, 449–518. Innocenzo Mazzini, ‘Caratteri della lingua del *De Medicina* di A. Cornelio Celso’, *Rivista di cultura classica e medioevale*, 1992, 34, 17–46. David Langslow, ‘Celsus and the Makings of a Latin Medical Terminology’, in Guy Sabbah and Philippe Mudry, eds, *La médecine de Celse: Aspects historiques, scientifiques et littéraires* (Saint-Étienne: Université de Saint-Étienne, 1994), 297–318.

48 Celsus, *Med.* proem.74–75. Translation from Walter Spencer, ed, *De Medicina, with an English Translation* (Cambridge, MA: Harvard University Press, 1938), I, 3. The compiler also copied the remaining few sentences of Celsus’ proem about the cruelty of vivisection and the importance of learning anatomy during dissections or while treating living patients.

49 For a description of hidden and evident causes, see Celsus, *Med.* proem.13–18.

50 Celsus, *Med.* proem.9. Ms Parisinus, 1v: ‘Cyrurgie vero inposuerunt anulum ferreum. Dietice [*sic*] quoque baculum propter quod ostendunt et philosophos in dracone. Farmaceutica pars est tertia propter quod multe experimentationes in erbis et in animalia essent’.

51 The other surviving late antique manuscript of Celsus’ *De medicina* is codex Florence, Biblioteca Medicea Laurenziana, Plut. 73.1. See Michael Reeve, ‘Celsus’, in Leighton Reynolds, ed, *Texts and Transmission* (Oxford: Clarendon Press, 1983), 46–47.

52 Ms Vat.lat. 5951, 87v, 90r. Ms Parisinus, 55v, 58v.

53 Ms Vat.lat. 5951, 85r. Ms Parisinus, 51v.

54 Ms Parisinus, 108r.

55 Celsus, *Med*. VI.5. Ms Parisinus, 48v-49r.

56 The text is partially edited in Daniël De Moulin, *De Heelkunde in de Vroege Middeleeuwen* (Leiden: Brill, 1964), 93–104. See also Henry Sigerist, ‘Die “Lecciones Heliodori”’, *Archiv für Geschichte der Medizin*, 1921, 13, 145–56. Klaus-Dietrich Fischer, ‘*“Universorum ferramentorum nomina.”* Frühmittelalterliche Listen chirurgischer Instrumente und ihr griechisches Vorbild’*, Mittellateinisches Jahrbuch*, 1987, 22, 28–44.

57 Celsus, *Med*. VII.proem.4-5.

58 See, for instance, manuscripts Florence, Biblioteca Medicea Laurenziana, Plut. 73.41, 122r-129v; London, British Library, Sloane 2839, 1v; Munich, Bayerische Staatsbibliothek, Clm 13002, 1v-2v.

59 Minta Collins, *Medieval Herbals: The Illustrative Traditions* (London: British Library, 2000), 183–4, 196. Pilsworth, *Healthcare*, 78–79.

60 Luis García-Ballester, ‘On the Origin of the Six Non-Natural Things in Galen’, in Luis García-Ballester *et al.*, eds, *Galen and Galenism: Theory and Medical Practice from Antiquity to the European Renaissance* (Burlington: Ashgate, 2002), 105–15. On late medieval understandings of air, see Craig Martin, ‘The Constitution of Air: Observation and the Limits of Temperament in Italian Renaissance Medical Writing’, *Early Science and Medicine*, 2023, 28, 560–62.

61 In the sixth century, Gregory of Tours rejected astrological predictions in favour of a ‘rational’ use of astronomy for computational purposes. Palmer, ‘Merovingian Medicine’, 42. See also Carine van Rhijn, ‘Review Article: Small Futures. Sortilege, Divination and Prognostication in Late Antiquity and the Early Middle Ages’, *Early Medieval Europe*, 2023, 31, 297–307.

62 On Carolingian diagrams, see Bruce Eastwood, *Ordering the Heavens: Roman Astronomy and Cosmology in the Carolingian Renaissance* (Leiden: Brill, 2007), 373–426.

63 Ms Parisinus, 154r. Jean Seznec, *The Survival of the Pagan Gods* (New York, NY: Pantheon Books, 1953), 63–64. Charles Clark, *The Zodiac Man in Medieval Medical Astrology* (Ann Arbor, MI: University Microfilms International, 1979), 171–72.

64 Edmond Liénard, ‘La mélothésie zodiacale dans l’Antiquité’, *Revue de l’Université de Bruxelles*, 1933, 39, 471–85. Faith Wallis, ‘Medicine in Medieval Calendar Manuscripts’, in Margaret Schleissner, ed, *Manuscript Sources of Medieval Medicine* (New York: Garland, 1995), 120. In the case of Parisinus, the correlation of signs and body parts does not match the most widely diffused ancient accounts, like the first-century CE *Astronomica* by Marcus Manilius. This specific correlation perhaps had a Greek source. The following list summarises the correlation of signs and bodily parts in codex Parisinus: Aries-forehead; Taurus-nostrils; Twins-eyes; Cancer-mouth; Leo-ears; Virgo-throat; Libra-chest; Scorpio-breast; Sagittarius-genitals; Capricorn-navel; Aquarius-thighs; Pisces-shins. See Clark, *The Zodiac Man*, 62–99, and the comparison tables in John Wee, ‘Discovery of the Zodiac Man in Cuneiform’, *Journal of Cuneiform Studies*, 2015, 67, 220–22.

65 Ms Parisinus, 154v-155v.

66 Ernest Wickersheimer, ‘Figures médico-astrologiques des IXe, Xe et XIe siècles’, *Janus*, 1914, 19, 164. Harry Bober, ‘The Zodiacal Miniature of the *Très Riches Heures* of the Duke of Berry: Its Sources and Meaning’, *Journal of the Warburg and Courtauld Institutes*, 1948, 11, 14–15. See also Barbara Obrist, ‘Le diagramme isidorien des saisons, son contenu physique et les représentations figuratives’, *Mélanges de l’école française de Rome*, 1996, 108, 95–164, and ‘La représentation carolingienne du zodiaque, à propos du manuscrit de Bâles, Universitätsbibliothek, F III 15a’, *Cahiers de civilisation médiévale*, 2001, 44, 10–11. Wolfgang Hübner, ‘La mélothésie zodiacale à la Renaissance’, in Véronique Dasen and Jean-Michel Spieser, eds, *Les savoirs magiques et leur transmission de l’Antiquité à la Renaissance* (Florence: SISMEL, Edizioni del Galluzzo, 2014), 304–6. John Murdoch, *Album of Science. Antiquity and the Middle Ages* (New York, NY: Scribner, 1984), 315–16.

67 Giuseppa Zanichelli, ‘“Philosophorum deliramenta”’, in Federica Toniolo and Gennaro Toscano, eds, *Miniatura. Lo sguardo e la parola* (Cinisello Balsamo: Silvana, 2012), 32–36.

68 Anastasia Panayotopoulou, ‘Roman Mosaics from Sparta’, *British School at Athens Studies*, 1998, 4, 112–18. Ruth Jacoby, ‘The Four Seasons in Zodiac Mosaics: The Tallaras Baths in Astypalaea, Greece’, *Israel Exploration Journal*, 2001, 51, 225–30. Rachel Hachlili, ‘The Zodiac in Ancient Jewish Synagogal Art: A Review’, *Jewish Studies Quarterly*, 2002, 9, 219–58, and *Ancient Mosaic Pavements* (Leiden: Brill, 2005), 35–56. Jodi Magness, ‘Heaven on Earth: Helios and the Zodiac Cycle in Ancient Palestinian Synagogues’, *Dumbarton Oaks Papers*, 2005, 59, 1–52. My thanks to Ishai Mishory for signalling the Beith Alpha mosaic.

69 Ms Vat.gr. 1291, 9r. Timothy Janz, ‘The Scribes and the Date of the Vat.gr. 1291’, *Miscellanea Bibliothecae Apostolicae Vaticanae*, 2003, 10, 159–80. See also Otto Neugebauer, ‘Melothesia and Dodecatemoria’, *Analecta Biblica*, 1959, 12, 270–75.

70 Wallis, ‘Calendar Manuscripts’, 116–17.

71 Murdoch, *Album of Science*, 343–45. Eastwood, *Ordering the Heavens*, 158–70.

72 Barbara Obrist, ‘Wind Diagrams and Medieval Cosmology’, *Speculum*, 1997, 72, 34 and 53–57. For an archaeological comparison, see the ‘Mosaic of the Charioteers’ in the Museo Nacional de Arte Romano, Merida, Spain. For similar diagrams in manuscripts, see ms Ghent, Universiteitsbibliotheek, 92, 24r, and ms Baltimore, The Walters Arts Museum, W.73, 1v.

73 Isidore, *On the Nature of Things* XXXVII and *Etymologies* XIII.xi.

74 Ms Parisinus, 161r-168v. On Athenagora’s *Liber de urinis*, see Arsenio Ferraces Rodrìguez, ‘*Liber Athenagore de urinis*, una traduccion latina de un compendio griego sobre semiotica de la orina’, in Anna Maria Urso, ed, *Il bilinguismo medico fra tardoantico e Medioevo* (Messina: EDAS, 2012), 89–90.

75 Ms Parisinus, 162r: ‘Animi an corporis vitio egritudo nata sit a se an extrinsecus evenerit, id est ex cibo vel potu aut ex corruptela aeris’.

76 Celsus, *Med.* II.1.10-16.

77 For a definition of formulas and proper recipes, see Gianna Pomata, ‘The Recipe and the Case. Epistemic Genres and the Dynamics of Cognitive Practice’, in Silvia Flurbacher, Kaspar von Greyerz, and Philipp Senn, eds, *Wissenschaftsgeschichte und Geschichte des Wissens im Dialog—Connecting Science and Knowledge* (Göttingen: V&R Unipress, 2013), 139–41.

78 On gynaecological recipes, see Monica Green, ‘The Development of the *Trotula*’, *Revue d’Histoire des Textes*, 1996, 26, 129, and *The Trotula: An English Translation of the Medieval Compendium of Women’s Medicine* (Philadelphia, PA: University of Pennsylvania Press, 2002). Early medieval gynaecological recipes are edited in Ferdinand Egert, *Gynäkologische Fragmente aus dem frühen Mittelalter* (Berlin: Ebering, 1936). On early medieval emmenagogues and abortifacients, see John Riddle, *Contraception and Abortion from the Ancient World to the Renaissance* (Cambridge, MA: Harvard University Press, 1992), 87–107.

79 Ms Parisinus, 158v (‘supponis in fisin’) and 159v (‘foveat in fisin’).

80 See the contributions by Eric Gowling, Laurence Totelin, and Gabrielle Lherminier in Lehmhaus and Martelli, eds, *Collecting Recipes*, 83–122 and 147–74. See also George Hourani, *Arab Seafaring in the Indian Ocean in Ancient and Early Medieval Times* (Princeton, NJ: Princeton University Press, 1951). Gerald Tibbetts, *A Study of the Arabic Texts Containing Material on South-East Asia* (Leiden: Brill, 1979). Axelle Rougeulle, ‘Medieval Trade Networks in the Western Indian Ocean (8th-14th centuries): Some Reflections from the Distribution Pattern of Chinese Imports in the Islamic World’, in Himanshu Prabha Ray and Jean-Francois Salles, eds, *Tradition and Archaeology: Early Maritime Contacts in the Indian Ocean* (New Delhi: Manohar Publishers and Distributors, 1996), 159–80. Anne McCabe, ‘Imported Materia Medica, 4th-12th Centuries, and Byzantine Pharmacology’, in Marlia Mundell Mango, ed, *Byzantine Trade, 4th-12th Centuries* (London: Routledge, 2009), 273–92.

81 John Riddle, ‘The Introduction and Use of Eastern Drugs in the Early Middle Ages’, *Sudhoffs Archiv für Geschichte der Medizin und der Naturwissenschaften*, 1965, 49, 194. Michael McCormick, *The Origins of the European Economy* (Cambridge: Cambridge University Press, 2001), 708–11.

82 On the tradition of drugs listing, see Nicholas Everett, *The Alphabet of Galen: Pharmacy from Antiquity to the Middle Ages* (Toronto: University of Toronto Press, 2012). For a detailed summary of the pre-modern pharmacopoeia, see Paula De Vos, *Compound Remedies: Galenic Pharmacy from the Ancient Mediterranean to New Spain* (Pittsburgh, PA: University of Pittsburgh Press, 2021), 36–62.

83 Riddle, ‘The Introduction and Use’, 185–98. McCormick, *The Origins*, 708–15. Paul Freedman, *Out of the East: Spices and the Medieval Imagination* (New Haven, CT: Yale University Press, 2008). Nicholas Everett, ‘The Manuscript Evidence for Pharmacy in the Early Middle Ages’, in Elina Screen and Charles West, eds, *Writing the Early Medieval West* (Cambridge: Cambridge University Press, 2018), 124–25. See also Claire Burridge, ‘Incense in Medicine: An Early Medieval Perspective’, *Early Medieval Europe*, 2020, 28, 219–55.

84 Zohar Amar and Efraim Lev, *Arabian Drugs in Early Medieval Mediterranean Medicine* (Edinburgh: Edinburgh University Press, 2017), 88–90 and 110–12.

85
*Ibid.*, 148–52 and 157–61. On musk, see Anna Akasoy, Charles Burnett, and Ronit Yoeli-Tlalim, *Islam and Tibet: Interactions along the Musk Routes* (Farnham: Ashgate, 2011). Anya King, *Scent from the Garden of Paradise. Musk and the Medieval Islamic World* (Leiden: Brill, 2017). On ambergris, see Freedman, *Out of the East*, 63–64, and Karl Dannenfeldt, ‘Ambergris: The Search for Its Origin’, *Isis*, 1982, 73, 382–97.

86 Ms Parisinus, 136r, 138v, 183r. On *cozumber* and *confita*, see Alejandro García González, ed, *Alphita* (Florence: SISMEL, Edizioni del Galluzzo, 2007), 174 and 184. The question of whether these substances’ appearance in recipes can be considered as conclusive evidence for established trade patterns remains open and subject to case-by-case study. The inclusion of new ingredients might suggest occasional, yet direct encounters with exotic substances, but recipes might also report latent knowledge that would become useful only according to the availability of ingredients. Burridge, *Carolingian Medical Knowledge*, 99–104.

87 Ms Parisinus, 186r-v. An eleventh-century version of the *diarhodon* is discussed in Florence Eliza Glaze, ‘Salerno’s Lombard Prince: Johannes “Abbas de Curte” as Medical Practitioner’, *Early Science and Medicine*, 2018, 23, 208–14.

88 See also the example of the ‘emplastrum barbara’ in ms Parisinus, 180v, as compared to Scribonius Largus, *Compositiones medicamentorum* 207.1 and Celsus, *Med.* V.19.

89 Lynn Thorndike, *History of Magic and Experimental Science* (New York, NY: Columbia University Press, 1923), I, 293. Robert Halleux and Jacques Schamp, eds, *Les lapidaires grecs* (Paris: Les Belles Lettres, 1985), XVI. Nicolas Weill-Parot, *Les “images astrologique” au Moyen Âge et à la Renaissance* (Paris: Honoré Champion, 2002), 109–12.

90 Julius Röhr, *Der okkulte Kraftbegriff im Altertum* (Leipzig: Dieterich, 1923), 95–113. Brian Copenhaver, *Magic in Western Culture: From Antiquity to the Enlightenment* (Cambridge: Cambridge University Press, 2015), 103–06.

91 Seznec, *The Survival*, 55–56. Erika Zwierlein-Diehl, ‘Magical Gems in the Medieval and Early-Modern Periods’, in Dasen and Spieser, eds, *Les savoirs magiques*, 87–130. John Riddle, ‘Lithotherapy in the Middle Ages... Lapidaries Considered as Medical Texts’, *Pharmacy in History*, 1970, 12, 39–50. Nichola Harris, ‘The Idea of Lapidary Medicine: Its Circulation and Practical Application in Medieval and Early Modern England, 1000-1750’ (unpublished PhD thesis, Rutgers University, 2009).

92 Ms Parisinus, 140v–143v.

93 Nicolas Weill-Parot, *Points aveugles de la nature: La rationalité scientifique médiévale face à l’occulte, l’attraction magnétique et l’horreur du vide* (Paris: Les Belles Lettres, 2013), 139–267. Christoph Sander, *Magnes: der Magnetstein und der Magnetismus in den Wissenschaften der Frühen Neuzeit* (Leiden: Brill, 2020), 532–6.

94 Ms Parisinus, 140r.

95 Tb 11:10-14. Galen and Dioscorides also argued that fish bile could heal leucoma, namely a white spot on the cornea. Bernd Kollmann, ‘Göttliche Offenbarung magisch-pharmakologischer Heilkunst im Buch Tobit’, *Zeitschrift für die alttestamentliche Wissenschaft*, 1994, 106, 289–99. See also Annie Attia, ‘Disease and Healing in the Book of Tobit and in Mesopotamian Medicine’, in Strahil Panayotov and Luděk Vacín, eds, *Mesopotamian Medicine and Magic* (Leiden: Brill, 2018), 36–68.

96 Kollmann, ‘Göttliche Offenbarung’, 291.

97 Peter Jones and Lea Olsan, ‘Performative Rituals for Conception and Childbirth in England, 900–1500’, *Bulletin of the History of Medicine*, 2015, 89, 406–33, esp. 407–09. Lea Olsan, ‘Charms and Prayers in Medieval Medical Theory and Practice’, *Social History of Medicine*, 2003, 16, 343–66. See also Maaike van der Lugt, ‘The Learned Physician as a Charismatic Healer: Urso of Salerno on Incantations in Medicine, Magic, and Religion’, *Bulletin of the History of Medicine*, 2013, 87, 307–46.

98 For a thorough discussion of early medieval theology, spirituality, and medicine, see Leja, *Embodying the Soul.* For early modern developments, see Lauren Kassell and Robert Ralley, ‘Prayer and Physic in Seventeenth-Century England’, *Early Science and Medicine*, 2021, 26, 480–508.

99 See also Sonja Brentjes, Alexander Fidora, and Matthias Tischler, ‘Towards a New Approach to Medieval Cross-Cultural Exchanges’, *Journal of Transcultural Medieval Studies*, 2014, 1, 9–50, esp. 23–24.

100 Laurence Totelin, *Hippocratic Recipes. Oral and Written Transmission of Pharmacological Knowledge in Fifth- and Fourth-Century Greece* (Leiden: Brill, 2009). Pomata, ‘The Recipe and the Case’. Elaine Leong, *Recipes and Everyday Knowledge: Medicine, Science, and the Household in Early Modern England* (Chicago, IL: University of Chicago Press, 2018).

101 See, for instance, Paula Findlen, *Possessing Nature. Museums, Collecting, and Scientific Culture in Early Modern Italy* (Berkeley, CA: University of California Press, 1994). Katie Whitaker, ‘The Culture of Curiosity’, in Nicholas Jardine, James Secord, and Emma Spary, eds, *Cultures of Natural History* (Cambridge: Cambridge University Press, 1996), 74–90. Brian Ogilvie, *The Science of Describing. Natural History in Renaissance Europe* (Chicago, IL: University of Chicago Press, 2006), 214–26.

## Notes

Silvia M. Marchiori is a PhD candidate in the Department of History and Philosophy of Science at the University of Cambridge. Her research explores the reception and circulation of ancient medical literature in medieval and early modern Europe, with a particular focus on Cornelius Celsus’ *De medicina*. Her doctoral project is supported by the Arts and Humanities Research Council through the Open-Oxford-Cambridge DTP [ref. AH/R012709/1], and she is the recipient of fellowships from the Folger, Huntington, and Osler Libraries, the New York Academy of Medicine, and the Gladys Krieble Delmas Foundation.

